# Familial *SYN1* variants related neurodevelopmental disorders in Asian pediatric patients

**DOI:** 10.1186/s12920-021-01028-4

**Published:** 2021-07-09

**Authors:** Juan Xiong, Haolin Duan, Shimeng Chen, Miriam Kessi, Fang He, Xiaolu Deng, Ciliu Zhang, Li Yang, Jing Peng, Fei Yin

**Affiliations:** 1 Department of Pediatrics, Xiangya Hospital, Central South University, No. 86, Xiangya Road, Kaifu District, Changsha, 410008 China; 2 Hunan Intellectual and Developmental Disabilities Research Center, Changsha, China

**Keywords:** *SYN1*, Variants, Neurodevelopmental disorders, Gender differences, Loss-of-function

## Abstract

**Background:**

*SYN1* encodes synapsin I, which is a neuronal phosphoprotein involving in regulating axonogenesis and synaptogenesis. Variants in the gene have been associated with X-linked neurodevelopmental disorders in recent years.

**Methods:**

In the study, we reported two male patients with familial *SYN1* variants related neurodevelopmental disorders from Asian population. Previously published cases with significant *SYN1* variants from the literature were also included to analyze the phenotype and genotype of the disorder.

**Results:**

Two maternally inherited *SYN1* variants, including c.C1076A, p.T359K in proband A and c.C1444T, p. Q482X in proband B (NM_133499) were found, which have never been described in detail. Combining with our research, all reported probands were male in the condition, whose significant *SYN1* variants were inherited from their asymptomatic or mild affected mother. Although the disorder encompasses three main clinical presentations: mental deficiency, easily controlled reflex seizure and behavior problems, patients’ clinical manifestations vary in genders and individuals, even in the same pedigree.

**Conclusion:**

We firstly reported two familial *SYN1*-related neurodevelopmental disorders in Asian pediatric patients. Gender and phenotype differences should be highly valued in the disorder.

## Background

SYN1(OMIM 313440) encodes Synapsin I protein, forming synaptic vesicles (SV) with other Synapsin subtypes including Synapsin II and Synapsin III, which has been confirmed to play crucial roles in synaptogenesis, synaptic neurotransmission, axonogenesis, and neuronal development in central and peripheral nervous system [1]. Homo sapiens Synapsin I protein has two isoforms, Ia (NP_008881, 705aa) and Ib (NP_598006, 669aa), both of which contain four former similar domains (A-D) and a distinctive C-terminus, with domain E in Synapsin Ia, domain F for Synapsin Ib [2]. Biallelic disruption of *SYN1* gene in mice results in seizures, autism-related behavioral abnormalities [3].

Variants in *SYN1* gene are related with X-linked epilepsy with variable learning disabilities and behavior disorders (OMIM 300491) and (or) X-linked intellectual disability (OMIM 300115) [4]. Like other X-linked neurodevelopmental disorders such as Turner-type X-linked syndromic mental retardation (OMIM 309590), the disease's clinical characteristics differ in gender. Most affected individuals are males, whose clinical presentations include variable degrees of intellectual disability/global developmental delay, epilepsy, movement disorder, autistic traits, and behavior problems. In contrast, female carriers are often asymptomatic or only exhibit mild cognitive impairment or febrile seizures [4]. All reported patients with *SYN1* related neurodevelopmental disorders are from European and North American studies, but none from Asian population.

In the study, we reported two maternally inherited *SYN1* variants (c.C1076A, p.T359K and c.C1444T, p. Q482X) (NM_133499) detected in two male pediatric patients with neurodevelopmental disorder. The study was accompanied by a comprehensive literature review about the genotypes, phenotypes of the condition. To the best of our knowledge, this is the first report about *SYN1*-related neurodevelopmental disorders in Asian population.

## Methods

### Subjects

Proband A and B were firstly referred to the Pediatric Department at Xiangya Hospital, Central South University with complaining of intellectual disabilities. Written informed consents for genetic testing were obtained from their pedigrees. The study was approved by the institutional medical ethics committee of Xiangya hospital, Central South Univesity.

### Molecular and bioinformatic analysis

Genomic DNAs were extracted from whole blood samples by using Phenol/chloroform method. The chromosomal karyotype and copy number variations of probands were detected by G-banding technique and chromosome microarray (CMA), separately. Trio whole-exome sequencing (Trio-WES) was performed on both pedigrees, and analyzed as described before [5]. Candidate variants were validated by Sanger sequencing. Variant impact predictor software including PolyPhen2 (http://genetics.bwh.harvard.edu/pph2/), PROVEAN (http://provean.jcvi.org/), Mutation Taster (http://www.mutationtaster.org/), and the American College of Medical Genetics (ACMG) guidelines, was used to analyze the pathogenicity of the variants [6].

### Literature review

Literature search on PubMed and Embase database in English was performed by combining “*SYN1*” or “Synapsin I” and “variants” or “mutations” until 24 April, 2021. Only those consumed significant *SYN1* variants were included to further analyze the genotypes of the disorder. Patients or pedigrees with clinical information were enrolled for concluding clinical features of *SYN1*-related neurodevelopmental disorder.

## Results

### Clinical information

#### Proband A and pedigree A

The patient was a 6 years and 3 months old boy, who was born in a non-consanguineous family. Before the birth of this child, his mother had a history of induced abortion due to taking fungal drugs, and a history of unexplained spontaneous abortion. In addition, the mother had an early threatened miscarriage during the pregnancy of the case, but got remissions after immediate medical intervention. The proband was delivered at full-term via caesarian section. The physical examination was normal at birth.

The most remarkable clinical symptom in proband A was profound global developmental delay since his infancy. His gross and fine motor skills were acquired and improved slowly: achieved neck muscle control at 15-month-old age, sat with support at 4-year-old age, walked dependently, but inflexible finger movements at the last evaluation. He also had great difficulties in language development. He only could make some responses to simple words or instructions, but had nonverbal expression at the last follow-up. His daily life needed to be fully taken care of by others. No behavior problem had been found in proband A until now. Two febrile convulsions were observed at his 1-year-old age and 2-year-old age. The electroencephalogram (EEG) and magnetic resonance imaging (MRI) of the brain at 4 years old age were normal. Meanwhile, the development quotients for adaptability, gross motor movements, fine motor movements, language, and individual–social interaction on the Gesell developmental scales were 36, 41, 30, 43, and 50, respectively. Other laboratory examinations, including the routine blood biochemical tests, metabolic analyses yielded negative. He had a medical history of bilateral esotropia.

**Fig. 1:**
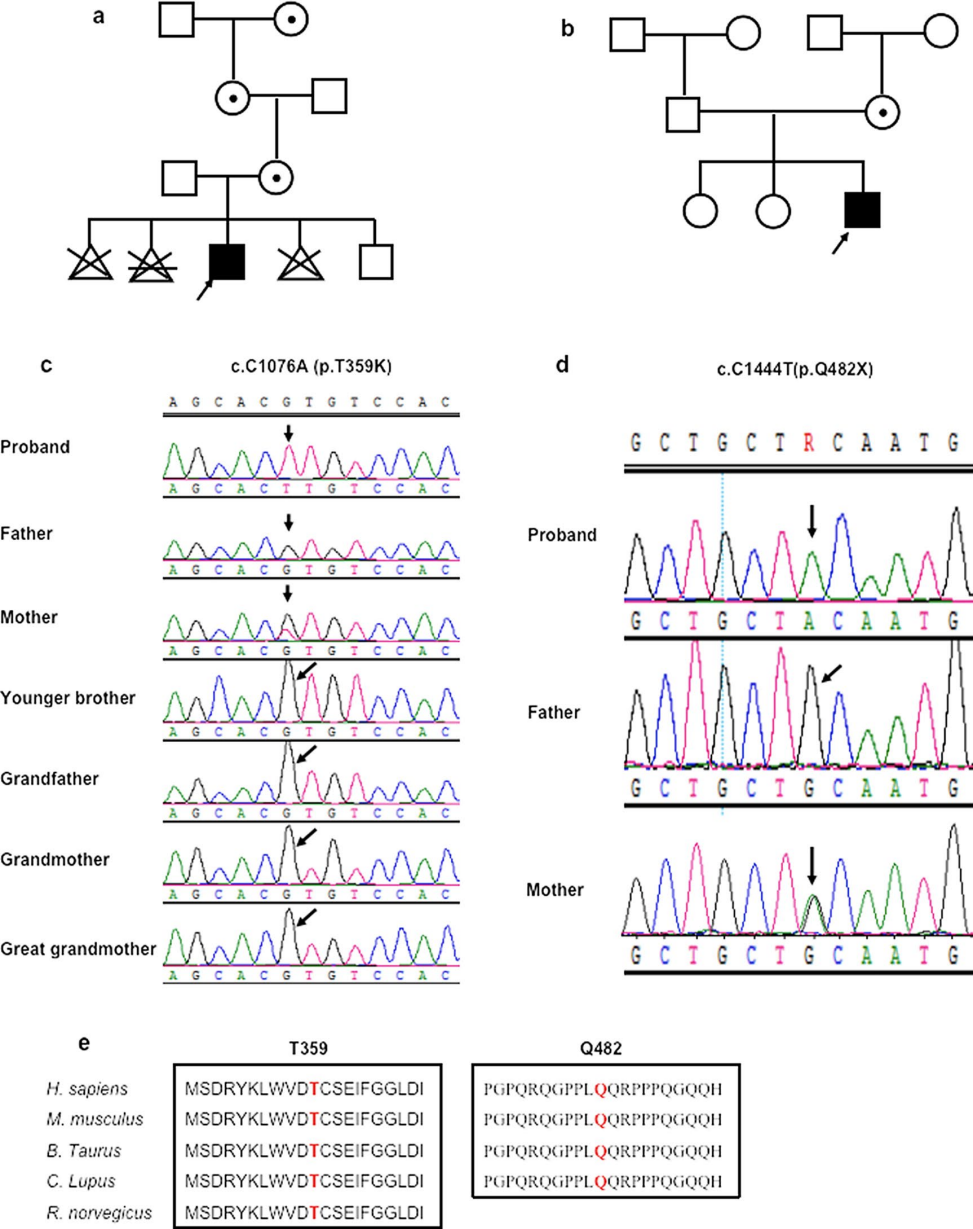
Two Asian patients with maternal inherited *SYN1* variants. The above two figures (**a**, **b**) show the segregation of the *SYN1* variants in the two pedigrees. **a** Pedigree A and **b** pedigree B:  , female carrier;  , affected male; arrow, the proband;  , induced abortion;  , spontaneous abortion. The middle two figures (**c**, **d**) show electropherograms of *SYN1* genomic sequences of the two pedigrees. The represented DNA sequences is in positive strand. **e** The alignment of the SynI protein sequence across species. The mutated amino acids are marked in red color, and lay in conserved positions as indicated

Proband A had a healthy younger brother whose age was two years old (Fig. 1a). His mother had no neurological signs. His biological father had self-limited febrile seizures at childhood. There was no other family history of neurological disorders to disclose.

### Proband B and pedigree B

An 8.5-year-old boy presenting with intellectual disability, epilepsy, abnormal social behavior, and ametropia came to our center's clinic. The patient was born as the third child of unrelated healthy parents after uneventful pregnancy and delivery. His distant uncle was diagnosed with intellectual disability, and an older female cousin had mood disorder.

The case began to have yearly epileptic seizures at his 7-year-old age, which was characterized with sudden loss of consciousness, rigidity of the limbs, and lips cyanosis, lasting for minutes. His video EEG revealed occasional sharp-waves in the bilateral frontal areas during sleep, that was absent in wake-time. Brain MRI showed no obvious structural abnormalities. His seizure was well controlled on levetiracetam at the last-follow-up. He lagged behind the peers in intelligence development, with poor school performance and bad communication skills. The intelligence quotient score at his 8.5-year-old age was 50.9. He also exhibited uncontrolled tempers, social problems, attention deficit, and hyperactivity. Nevertheless, the behavior assessments, including Conners' Comprehensive Behavior Rating Scales (CBRS), Kiddie-SADS DSM-5 Screen Interview (K-SADS-PL), and Autism Behavior Checklist (ABC) were negative. Metabolic, immunological, and infectious etiology were excluded after the diagnostic workup.

The proband had two healthy elder sisters, 18 and 14 years old. They both performed well in school (Fig. 1b). His low-level educated parents could work and support their family well.

### Genetic findings

The analyses of karyotype and copy number variation in the two probands showed no abnormalities in their chromosomes. Maternal inherited *SYN1* variants were identified by Trio-WES.

In pedigree A, the missense variant c.C1076A, p.T359K in exon 9 of *SYN1* gene (NM_133499) was present in a hemizygous state in proband A and a heterozygous state in his mother, grandmother, and great-grandmother as obligate carriers, but was absent in his younger brother, father, and grandfather (Fig. 1a, c). The variant T359K is recorded as a variant of uncertain significance in ClinVar database records (accession VCV000589101.2), associated with a "history of neurodevelopmental disorder" [7]. And 1 of 161,942 individuals in the gnomAD database showed hemizygous presence of variant T359K [8]. The substitution of lysine for threonine acid at position 359 within domain C of SYN1 protein is considered as "possibly damaging" (score 0.886) with PolyPhen2 (http://genetics.bwh.harvard.edu/pph2/), "deleterious" (score − 2.565) with PROVEAN (http://provean.jcvi.org/) and "disease-causing" (score 78) with MutationTaster (http://www.mutationtaster.org/).

In pedigree B, the truncated variant c.C1444T, p.Q482X (NM_133499) in exon 12 of *SYN1* was identified, which was present in a hemizygous state in the proband, and a heterozygous state in his mother, but was absent in his father (Fig. 1d). The variant validation of other family members was not done for financial reasons (Fig. 1b). The variant has never been reported before and could not be found in dbSNP143, gnomAD, and Clinvar database. The nonsense variant Q482X lies in the domain D of the protein, and is identified as "disease-causing" (score 6) with MutationTaster (http://www.mutationtaster.org/).

Bioinformatic analysis showed that sites T359 and Q482 are highly conserved residues among different species (Fig. 1e). The variant T359K was classified as “Uncertain significance” (PM1 + PM2 + PP3), while the other variant was “Pathogenic” (PVS1 + PM2 + PP3), according to the ACMG guidelines [6].

### Genotype and phenotype of *SYN1*-related neurodevelopmental disordrer

**Fig. 2:**
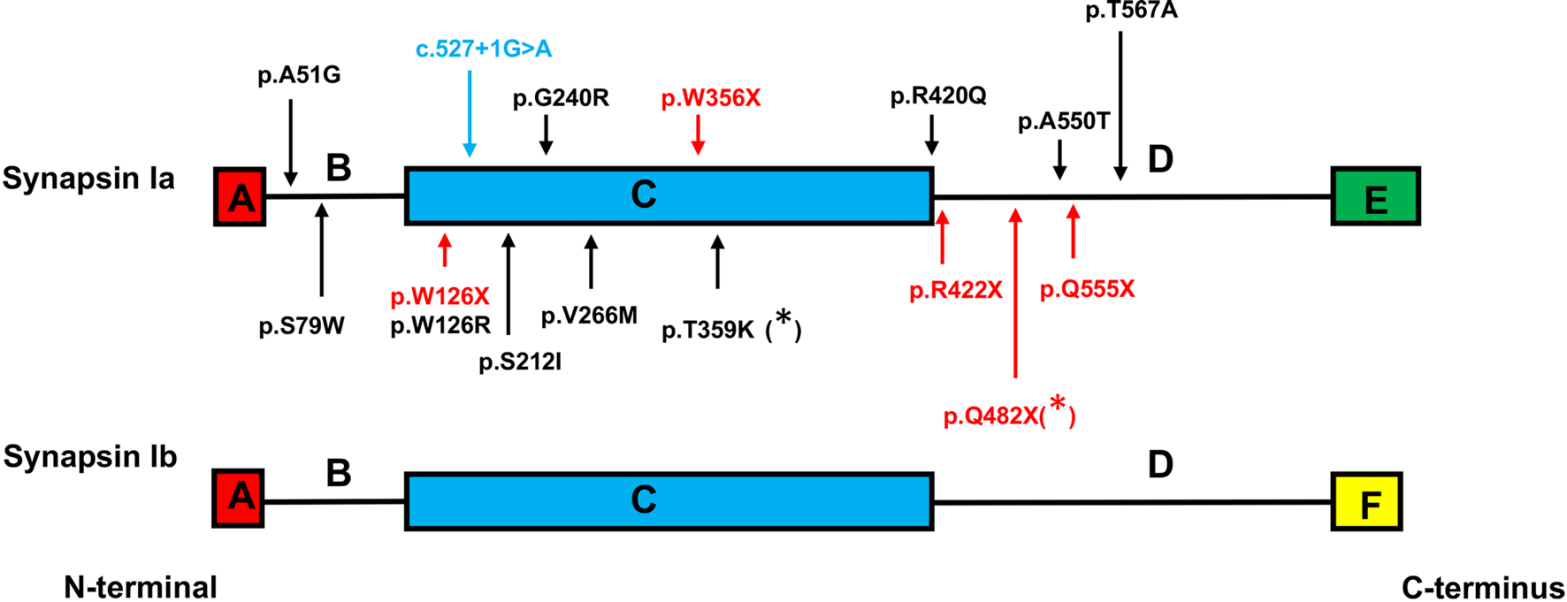
Synapsin I protein diagram and the distribution of the variants. The *SYN1* gene encodes two isoforms, Ia and Ib. They have four former similar domains (A**–**D), and a distinctive C-terminus (domain E or F). The isoform of Synapsin Ia is longer and expressed higher than Synapsin Ib. The variants identified in previous studies and this study (marked as asterisk) are indicated with different colors denoting different mutation types, including 10 missense variants (in black color), 5 truncation variants (in red color), and 1 splicing variant (in blue color)

Since Garcia CC et al. firstly connected *SYN1* variants with neurodevelopmental disorder in 2004, 16 causative variants including ten missense mutations, five nonsense mutations, and one splicing site mutation in the gene have been reported (containing this study) (Fig. 2) [1, 4, 9–18]. These variants are clustered in B linker domain (A51G, S79W), actin-binding and synaptic-vesicle binding C-domain (W126X, W126R, c.527 + 1G > A, S212I, G240R, V266M, W356X, T359K, R420G) and proline-rich D-domain (R422X, Q482X, A550T, Q555X, T567A) of synapsin I as indicated (Fig. 2).

Clinical information of 7 pedigrees in 7 reported studies and 2 pedigrees in our study was summarized in Table 1 [4, 11–16], after excluding those cases without detailed clinical descriptions [1, 9, 10, 17, 18]. 5/9 pedigrees had normal or mild cognitive impairments, 3/9 pedigrees had moderate to severe global developmental delay, while 1/9 pedigree showed mental regression (Table 1). Cases in 6/9 pedigrees were diagnosed with epilepsy, which could be triggered by bathing or showering [11, 15]. Most cases had mild seizure forms which responded well to antiepileptic drugs. Behavioral problems such as aggression, hyperactivity, and autistic traits were reported in 6/8 pedigrees.

**Table 1:** Clinical and genetic characteristics of patients with SYN1-related disorders in literature and the study

References	Garcia et al. [4]	Nguyen et al. [11]	Sirsi et al. [12]*	Guarnieri et al. [16]	Peron et al. [15]	Darvish et al. [14]	Ibarluzea et al. [13]	Pedigree A (this study)	Pedigree B (this study)
Country (ethnic)	England (unknown)	Canada (French-Canadian)	﻿America (Latino)	Italy (unknown)	Italy (unknown)	America (unknown)	Spain (unknown)	China (Han)	China (Han)
*Basic information*									
Sex of probands	Male (assumed)	Male	Male	Unknown	Male	Male	Male	Male	Male
Affected family members	Male (10)	Male (10); Female (2)	–	Male (8); Female (2)	Male (2)	Male (3)	Male (2)	Male (1)	Male (1)
Carrier family members	Female (9)	Female (8)	Female (1)	Female (1)	Female (1)	Unknown	Female (5)	Female (3)	Female (1)
*SYN1* variants	c.G1068A (p.W356X) [NM_133399]	c.C1663T (p.Q555X) [NM_133399]	﻿c.C1264T (p.R422X) [NM_133399]	c.C236G (p.S79W) [NM_133399]	c.527 + 1G > T [NM_133399]	c.G1259A (p. R420Q) [NM_133399]	c.G796A (p.V266M) [NM_133399]	c.C1076A (p.T359K) [NM_133399]	c.C1444T (p.Q482X) [NM_133399]
Diagnosis	Variable epilepsy, learning disabilities, and aggressive behavior	﻿﻿X-linked focal epilepsy with reflex related-bathing seizures	﻿Focal epilepsy and reflex related- bathing seizures, autism, and intellectual disability	Non-syndromic intellectual disability	Hot water-sensitive epilepsy	Autism and progressive intellectual disability without epilepsy	Intellectual disability and paranoid schizophrenia	Intellectual disability and complex febrile seizures	Epilepsy, behavioral disorders and learning disabilities
*Clinical features*									
Degree of intellectual disability	Normal or mild	Normal or mild	–	Moderate to severe	Normal or mild	ID from early childhood mental regression	Mild	Profound	Moderate
Presence of epilepsy	Y	Y	Y	N	Y	N	N	Y	Y
Onset age of seizures	6–27y	1y8m–50y	Early childhood	–	8y	-	-	1y	7y
Seizure semiology	Tonic–clonic, reflex, and partial and complex-partial seizures	Spontaneous complex partial seizures and reflex seizures triggered by bathing	Focal seizures and reflex seizures triggered by bathing	–	Hot water sensitive seizures at the beginning, subsequently followed by nonreflex seizures	-	-	Tonic–clonic seizures triggered by fever	Tonic–clonic seizures
Seizure frequency	Episodic	Episodic	1–2 times per month	–	Unknown	-	-	Only 2 times	Episodic
Seizure control or not	Y	Most affected members have achieved seizure control	﻿Intractable to AEDs, seizures reduce about 50% by VNS	–	Unknown	–	–	Y	Y
*Abnormal behavior*									
Aggression	Y	N	N	–	N	N	N	N	Y
Autistic traits	Y	Y	Y	–	N	Y	Y	N	N
EEG findings	Some evidence of spikes in the left temporal region or normal	Rhythmic theta activity over temporal head regions	﻿Spikes on the left temporal region	–	Bilateral rhythmic theta activity over the frontocentral and vertex regions	–	–	Normal	Occasional sharp-waves occurring in bilateral frontal areas during sleep
Brian MRI imaging	Normal	Hippocampal atrophy	Normal	–	–	Marked generalized frontal atrophy	–	Normal	Normal
Other findings	Macrocephaly	N	N	N	N	Sphincter dysfunction	N	Bilateral esotropia	Ametropia

*AEDs* anti-epileptic drugs,* EEG* electroencephalography,* ID* intellectual disability,* MRI* magnetic resonance imaging,* m* month,* N* no,* Y* yes,* y* year,* VNS* vagal nerve stimulator

*The pedigree in the study has a maternal family history of epilepsy but lacks familial genetic results due to financial reasons

## Discussion

*SYN1* is the only member of the synapsin gene family that is confirmed as pathogenic cause of human monogenic disease until now. Notably, all significant *SYN1* variants in literature and our research were confirmed to be inherited from the maternal side, supporting a characteristic of familial clustering in the disease. Therefore, detailed family history inquiries would help clinical diagnosis. Interestingly, none of identified causative *SYN1* variants located in domain A and E or F of the encoded protein. But some *SYN1* variants in these domains were recorded in the gnomAD database with low-rate frequency [8]. Domain A is highly conserved in diverse synapsin subtypes and species [2], and domain E or F play functions in forming SV reserve pool, regulating kinetics of exocytosis, and SV cycling [2, 19]. We speculated that defects in the middle motifs of Synapsin I, including domain B, C and D, are inclined to lead to neurodevelopmental disorders in human after birth, while the possible harmfulness of variants in the other domains remain unknown.

Here, we firstly reported two male patients with *SYN1*-related neurodevelopmental disorder in Asian population, which added clinical evidence for the condition. Although proband A had more impaired cognition than most cases in the disease, Darvish et al. also identified three male patients with progressive intellectual disabilities in an American family with significant *SYN1* variant [14]. Patient A had an uncertain significance *SYN1* variant (c.C1076A, p.T359K), which was not present in his healthy brother, and the Trios-WES showed no other possible pathogenic variants. Therefore, in the present situation, we considered the variant could explain proband A’s clinical symptoms, functional studies might help us make decisions in future research. Moreover, proband A was more in line with the diagnosis of X-linked intellectual disability. For proband B, he had a pathogenic *SYN1* variant. He showed mild cognition impairment, learning disabilities and behavior problems, which was a typical case of X-linked epilepsy with variable learning disabilities and behavior disorders.

In the other hand, the phenotypes of the disease differ in genders and individuals. Overall, we noticed that females are less susceptible than males, and only few female carriers present mild cognition impairment and febrile seizures, which might attribute to X-chromosome random inactivation (XCI) [10, 16]. And probands in previous reported pedigrees often are male, suggesting that female patients are prone to be neglected. In our study, only male patients had clinical manifestations, while female obligate carriers had no neurological signs. It is worth noting that these phenotypic differences of this disease also exist in different individuals of the same sex in the same pedigree. In an England four generation family, 10 male patients were identified. Some of them only had epilepsy, while others also had learning difficulties, macrocephaly, and aggressive behavior [4]. The phenotypic differences among different individuals in the disorder may be due to the complementary mechanism of synaptic protein functions in the body, which needs further research. Hence, we reached an conclusion that *SYN1*-related disorder is a neurodevelopmental disorder with high clinical heterogeneity.

However, we still could conclude three main clinical manifestations of the condition after systematic reviewing previous reported cases with *SYN1*-related disorder. Firstly, patients had variable degrees of intellectual disabilities. As was described in the result section, normal or mild cognition impairments were more common in these pedigrees. The reflex seizure related to bathing or showering, was another remarkable clinical manifestation in the disorder [11, 15]. Our cases had no history of reflex seizure, but proband A was diagnosed as febrile seizures. Their seizures often responded well to antiepileptic drugs. Behavioral problems such as aggression, hyperactivity, and autistic traits were the third typical clinical features of *SYN1*-related disorders. Proband B in the research had apparent aggressive behaviors and attention-deficit, even though his behavioral assessment scales were negative. Patients with the above three significant clinical symptoms need to be alert to the possibility of *SYN1*-related neurodevelopmental disorder.

Moreover, we also noticed that our two cases have eye problems including ametropia and strabismus, which has not been mentioned in other studies. According to the Human protein atlas database, SYN1 is expressed in human eye tissue at RNA level, nevertheless, there is no further evidence to support involvement of the protein in eye development [20]. Eye disorder might be a new clue for clinicians to identify the condition, however, it needs more data.

Several functional analyses have been done in recent studies to elucidate the possible pathogenesis mechanisms in *SYN1*-related disorder [1, 14, 16]. Compared to human *SYN1* wildtype, expression of human *SYN1* variants such as S79W, A550T, T567A in *SYN1* knockout (KO) mouse neurons failed to rescue neuron cell size and SV pools trafficking, and resulted in defective nerve terminal function [1, 16]. Variant R420Q could significantly disrupted neurite outgrowth and development in mouse primary hippocampal neurons [14]. These findings implicate that *SYN1* variants detected in neurodevelopmental disorders lead to loss-of-function of the Synapsin-I protein in the brain network, and patients with the disease might benefit from improved protein function.

## Conclusion

In conclusion, this is the first study about *SYN1*-related neurodevelopmental disorder in Asian population, which expands the genetic spectrum of the disease. Remarkably, all reported significant *SYN1* variants were maternally inherited, and located in middle domains of the gene. Gender differences and phenotype variances should be considered in the disorder. Besides mental deficiency, reflex seizures and behavior problems, eye disorders might be helpful to identify the condition. Combining with previous studies, loss-of-function of Synapsin-I protein is the possible mechanism in patients with the disorder.

## Data Availability

All data supporting our results can be found in a published article. Current data on patients cannot be fully accessible in accordance with local research ethics protocols. However, if you are interested in this article, it may be available from the corresponding author.

## Abbreviations

ACMG: American College of Medical Genetics
*SYN1*: *Synapsin I*
OMIM: Online mendelian inheritance in man
CMA: Chromosome microarray
Trio-WES: Trio whole-exome sequencing
EEG: Electroencephalogram
MRI: Magnetic resonance imaging
CBRS: Conners' Comprehensive Behavior Rating Scales
K-SADS-PL: Kiddie-SADS DSM-5 Screen Interview
ABC: Autism Behavior Checklist
SV: Synaptic vesicle
XCI: X-chromosome random inactivation
RNA: Ribonucleic acid
KO: Knockout
